# Ten quick tips for creating an effective lesson

**DOI:** 10.1371/journal.pcbi.1006915

**Published:** 2019-04-11

**Authors:** Greg Wilson

**Affiliations:** RStudio, Toronto, Ontario, Canada; University of Toronto, CANADA

## Abstract

We present 10 tips for building effective lessons that are grounded in empirical research on pedagogy and cognitive psychology and that we have found to be practically useful in both classroom and free-range settings

## Author summary

As a species, we know as much about teaching and learning as we do about public health, but most people who teach at the postsecondary level are never introduced to even the basics of evidence-based pedagogy. Knowing just a few key facts will help you build more effective lessons in less time and with less pain and will also make those lessons easier for your peers to find and reuse. This paper presents 10 tips that you can apply immediately and explains why they work.

## Introduction

There are many kinds of lessons, both formal and informal, from seconds long to lifelong. Most people have sat (or suffered) through hundreds of these but have never been shown how to design ones that are effective. These 10 simple tips for creating lessons are

- based on current educational research [1, 2, 3, 4, 5, 6],
- filtered by what can be done by nonspecialists with limited time and resources [7, 8], and
- prioritized by experience teaching and training people to teach together [9, 10, 11].

The key insight that underpins all of these tips is that learning is both a cognitive and a social activity. On the cognitive side, incoming information (the lesson) passes through a “sensory register” that has physically separate channels for visual and auditory information and is stored in short-term memory, where it is used to construct a “verbal model” (sometimes also called a “linguistic model”) and a separate “visual model” [12]. These are then integrated and stored in long-term memory as facts and relationships. If those facts and relationships are strengthened by use, they can later be recalled and applied, and we say that learning has taken place.

One key feature of this model is that short-term memory is very limited: [13] famously estimated its size as 7 ± 2 items, and more recent studies place the figure closer to 4. If too much information is presented too quickly, material spills out of short-term memory before it can be integrated and stored, and learning does not occur.

A second key feature is that the brain's processing power is also very limited. Effort spent identifying key facts or reconciling the linguistic and visual input streams reduces the power available for organizing new information and connecting it to what's already present.

Learning is also a social activity. Learners who feel motivated will learn more; learners who feel that they may not be judged on their merits or who have experienced unequal treatment in the past will learn less (see the tip "Motivate and avoid demotivating"). In [14], e.g., Kenneth Wesson wrote, "If poor inner-city children consistently outscored children from wealthy suburban homes on standardized tests, is anyone naive enough to believe that we would still insist on using these tests as indicators of success?" Lesson designers must take the social aspects of learning into account if they are to create effective lessons; we discuss this further in the final tip ("Make lessons inclusive").

## 1. Use learner personas to define your audience

The first step in creating a good lesson is figuring out who the audience is. One way to do this is to make up biographies of two or three target learners. This technique is borrowed from user interface designers, who create short profiles of typical users to help them think about their audience.

These profiles are called “personas” and have five parts:

1. the person's general background,
2. what they already know,
3. what they think they want to do (as opposed to what someone who already understands the subject thinks),
4. how the lesson will help them, and
5. any special needs they might have.

A learner persona for a weekend introduction to programming aimed at college students might be as follows:

1. Jorge has just moved from Costa Rica to Canada to study agricultural engineering. He has joined the college soccer team and is looking forward to learning how to play ice hockey.
2. Other than using Excel, Word, and the internet, Jorge's most significant previous experience with computers is helping his sister build a WordPress site for the family business back home in Costa Rica.
3. Jorge needs to measure properties of soil from nearby farms using a handheld device that sends logs in a text format to his computer. Right now, Jorge has to open each file in Excel, crop the first and last points, and calculate an average.
4. This workshop will show Jorge how to write a little Python program to read the data, select the right values from each file, and calculate the required statistics.
5. Jorge can read English well but still struggles sometimes to keep up with spoken conversation (especially if it involves a lot of new jargon).

Rather than writing new personas for every lesson or course, instructors often create and share a handful that cover everyone they hope to teach, then pick a few from that set to describe who particular material is intended for. Used this way, personas become a convenient shorthand for design issues: when speaking with each other, teachers can say, "Would Jorge understand why we're doing this?" or, "What installation problems would Jorge face?"

Personas help you remember one of the most important tips of teaching: you are not your learners. The people you teach will almost always have different backgrounds, different capabilities, and different ambitions than you; personas help you keep your lessons focused on what they need rather than on what your younger self might have wanted.

## 2. Design for effective learning strategies

Some learning strategies are provably more effective than others [15, 16, 17], so lessons should be designed to encourage their use. As summarized in [18, 19], the six most important are as follows:

- Ten hours of study spread out over 5 days are more effective than two 5-hour days and far better than one 10-hour day. You should therefore create lessons and exercises that include some older material in each new lesson. According to [20], "The lectures that predominate in face-to-face courses are relatively ineffective ways to teach, but they probably contribute to spacing material over time, because they unfold in a set schedule over time. In contrast, depending on how the courses are set up, online students can sometimes avoid exposure to material altogether until an assignment is nigh."
- Researchers now believe that the limiting factor for long-term memory is not retention (what is stored) but recall (what can be accessed). Recall of specific information improves with practice, so outcomes in real situations can be improved by taking practice tests or summarizing the details of a topic from memory and then checking what was and wasn't remembered. [21], e.g., found that repeated testing improved recall of word lists from 35% to 80%.
- One way to space retrieval practice is to interleave study of different topics: instead of mastering one subject, then the next, then a third, shuffle the order. Even better, switch up the order: A-B-C-B-A-C is better than A-B-C-A-B-C, which in turn is better than A-A-B-B-C-C [15]. This is effective because interleaving fosters creation of more links between different topics, which in turn increases retention and recall.
- Having learners explain things to themselves and others as they go along improves understanding and recall. One way to do this is to follow up each answer on a practice quiz with an explanation of why that answer is correct or, conversely, with an explanation of why some other plausible answer isn't. Another is to have learners explain how a new idea is similar to or different from one they have seen previously.
- One specific form of elaboration that is useful enough to deserve its own heading is the use of concrete examples. As discussed in the tip "Use worked examples and concreteness fading," every statement of a general principle should be accompanied by one or more examples of its use or, conversely, should take each particular problem and list the general principles it embodies. [22] found that interleaving examples and definitions made it more likely that learners would remember the latter correctly.
- Another approach is to teach by contrast, i.e., to show learners what a solution is not or what kind of problem a technique won't solve. When showing children how to simplify fractions, e.g., it's important to give them a few like 5/7 that can't be simplified so that they don't become frustrated looking for answers that don't exist.

Different subsystems in our brains handle and store linguistic and visual information, and if complementary information is presented through both channels, then they can reinforce one another. However, learning is more effective when the same information is not presented simultaneously in two different channels [23, 12] because then the brain has to expend effort to check the channels against each other. This is one of the many reasons that reading slides verbatim is ineffective: not only is the reader not adding value, they are actually adding to the load on learners whose brains are trying to check that the spoken and written inputs are consistent.

## 3. Write summative assessments to set concrete goals

“Summative assessment” is something done at the end of a lesson to tell whether the desired learning has taken place: a driving test, performance of a piece of music, a written examination, or something else of that kind. Summative assessments are usually used as gates (e.g., "Is it now safe for this person to drive on their own?"), but they are also a good way to clarify the learning objectives for a lesson. "Understand linear regression" is hopelessly vague; a much better way to set the goal for that lesson would be to define an exercise, such as the following:

Write a short R script that reads the tabular data in housing.csv and uses the lm function to calculate a regression coefficient relating house price to purchaser age.

This is better because it gives the lesson author a concrete goal to work toward: nothing goes in the lesson except what is needed to complete the summative assessments. This helps reduce content bloat and also tells the author when the lesson is done.

Writing summative assessments early in the lesson design process also helps ensure that outcomes are actually checkable. Since telepathy is not yet widely available, it is impossible for instructors to know what learners do and don't understand. Instead, we must ask them to demonstrate that they're able to do something that they couldn't do without the desired understanding.

Finally, creating summative assessments early can help authors stay connected to their learners' goals. Each summative assessment should embody an “authentic task,” i.e., something that an actual learner actually wants to do. Early on, authentic tasks should be learners' own goals; as they advance and are able to make sense of generalizations, these tasks may be extensions or generalizations of earlier solutions.

Continuing with the statistical example above, calculating a regression coefficient may be an authentic task for someone who already knows enough statistics to understand what such coefficients are good for. If the intended learners are not yet that experienced, this exercise could be extended to have them make some sort of judgment based on the regression coefficients to exercise higher-order thinking.

## 4. Write formative assessments for pacing, design, preparation, and reinforcement

The counterpoint to summative assessment is “formative assessment,” which is checks that are used while learning is taking place to form (or shape) the teaching. Asking learners for questions is a common, but relatively ineffective, kind of formative assessment. What works better is to give them a short problem—one that can be done in 1–2 minutes so as not to derail the flow of the lesson and that will help them uncover and confront their misconceptions about the topic being taught.

Checking in with learners this way every 10–15 minutes accomplishes several things:

- Asking "Does everyone understand?" almost always produces false positives. In contrast, if any substantial fraction of your learners cannot do a formative assessment correctly, you know right then and there that you need to re-explain the most recent material. When you start doing this, you will feel like you're going more slowly, but that's because you will now be teaching at the speed at which your audience can learn rather than the speed at which you can talk.
- Creating formative assessments that build toward a lesson's summative assessment gives you a structure for your lesson. Returning to the regression example, the summative assessment tells you that you should have exercises along the way in which learners load CSV data, use the lm function with appropriate parameters, and interpret the result. Writing a few minutes of material for each of these subjects is less intimidating than trying to explain the whole topic at once.
- Formative assessments give learners practice with the concepts, methods, and tools they will use when doing the lesson's summative assessment and tells them where to focus their revision. Switching from statistics to music, if a violinist is able to do the bowing and fingering exercises for a piece but is struggling with the rhythmic patterns, that tells her where she should spend her study time.
- Learners remember things better if they use material right away; having formative assessments during the lesson does this.
- Breaking a summative assessment down into parts and creating formative assessments for each usually shows you that you are trying to cram too much into one lesson. Writing assessments is therefore iterative, as early drafts of summative assessments are rescoped to only require as much material as can plausibly be covered.

[24, 25, 26] offer inspiration for a wide variety of different kinds of summative and formative assessment exercises.

## 5. Integrate visual and linguistic information

Research by Mayer and colleagues on the split-attention effect is closely related to cognitive load theory [23]. As described in the introduction, linguistic and visual input are processed by different parts of the human brain, and linguistic and visual memories are stored separately as well. This means that correlating linguistic and visual streams of information takes cognitive effort: when someone reads something while hearing it spoken aloud, their brain can't help but check that it's getting the same information on both channels.

Learning is therefore more effective when information is presented simultaneously in two different channels, but when that information is complementary rather than redundant. People generally find it harder, e.g., to learn from a video that has both narration and on-screen captions than from one that has either the narration or the captions but not both because some of their attention has to be devoted to checking that the narration and the captions agree with each other. Two notable exceptions to this are people who do not yet speak the language well and people with hearing exercises or other special needs, both of whom may find that the extra effort is a net benefit.

This is why it's more effective to draw a diagram piece by piece while teaching rather than to present the whole thing at once. If parts of the diagram appear at the same time as things are being said, the two will be correlated in the learner's memory. Pointing at part of the diagram later is then more likely to trigger recall of what was being said when that part was being drawn.

The split-attention effect does not mean that learners shouldn't try to reconcile multiple incoming streams of information—after all, this is something they have to do in the real world [27]. Instead, it means that instruction shouldn't require it while people are mastering unit skills; instead, using multiple sources of information simultaneously should be treated as a separate learning task.

## 6. Design for peer instruction

No matter how good a teacher is, she can only say one thing at a time. How then can she clear up many different misconceptions in a reasonable time? The best solution developed so far is peer instruction. Originally created by Eric Mazur at Harvard [28], it has been studied extensively in a wide variety of contexts (e.g., [29, 30]).

Peer instruction is essentially a scalable way to provide one-to-one mentorship. It interleaves formative assessment with student discussion as follows:

1. Give a brief introduction to the topic, either in class or in out-of-class reading.
2. Give learners a multiple-choice question (MCQ).
3. Have all the students vote on their answers to the MCQ.
  - If all the students have the right answer, move on.
  - If they all have the same wrong answer, address that specific misconception.
  - If they have a mix of right and wrong answers, give them several minutes to discuss those answers with one another in small groups (typically 2–4 students) and then reconvene and vote again.

The questions posed to learners don't have to be MCQs: matching terms to definitions can be equally effective, as can Parsons Problems (in which they are given the jumbled parts of a solution and must put them in the right order [31]). Whatever mix is used, the lesson must build toward them, and the question must probe for conceptual understanding and misconceptions (rather than check simple factual knowledge).

Group discussion significantly improves students' understanding because it forces them to clarify their thinking, which can be enough to call out gaps in reasoning. Repolling the class then lets the teacher know whether they can move on or whether further explanation is necessary. A final round of additional explanation and discussion after the correct answer is presented gives students one more chance to solidify their understanding.

But could this be a false positive? Are results improving because of increased understanding during discussion or simply from a follow-the-leader effect? [32] tested this by following the first question with a second one that students answer individually and found that peer discussion actually does enhance understanding, even when none of the students in a discussion group originally knew the correct answer.

It is important to have learners vote publicly so that they can't change their minds afterwards and rationalize it by making excuses to themselves like “I just misread the question.” Some of the value of peer instruction comes from having their answer be wrong and having to think through the reasons why. This is called the “hypercorrection effect” [33]. Most people don't like to be told they're wrong, so it's reasonable to assume that the more confident someone is that the answer they've given in a test is correct, the harder it is to change their mind if they were actually wrong. However, it turns out that the opposite is true: the more confident someone is that they were right, the more likely they are not to repeat the error if they are corrected.

## 7. Use worked examples and concreteness fading

A worked example is a step-by-step demonstration of how to solve a problem or do some task. By giving the steps in order, the instructor reduces the learner's cognitive load, which accelerates learning [27, 34].

However, worked examples become less effective as learners acquire more expertise [35, 36], a phenomenon known as the “expertise reversal effect.” In brief, as learners build their own mental models of what to do and how to do it, the detailed step-by-step breakdown of a worked example starts to get in the way. This is why tutorials and manual pages both need to exist: what's appropriate for a newcomer is frustrating for an expert, while what jogs an expert's memory may be incomprehensible to a novice.

One powerful way to use worked examples is to present a series of “faded examples” [37]. The first example in the series is a complete use of a problem-solving strategy; each subsequent example gives the learner more blanks to fill in. The material that isn't blank is often referred to as scaffolding since it serves the same purpose as the scaffolding set up temporarily at a building site.

Faded examples can be used in almost every kind of teaching, from sports and music to contract law. Someone teaching high school algebra might use them by first solving this equation for *x*:

(4*x* + 8)/2 = 5
    4*x* + 8 = 2 * 5
    4*x* + 8 = 10
        4*x* = 10–8
        4*x* = 2
            *x* = 2/4
            *x* = 1/2

and then asking learners to fill in the blanks in this:

(3*x* – 1) * 3 = 12
    3*x* – 1 = __/__
    3*x* – 1 = 4
        3*x* = __
            *x* = __/3
            *x* = __

The next problem might be this:

(5*x* + 1) * 3 = 4
    5*x* + 1 = __
        5*x* = __
            *x* = __

Learners would finally be asked to solve an equation entirely on their own:

(2x+7)/4=1

At each step, learners have a slightly larger problem to solve, which is less intimidating than a blank screen or a blank sheet of paper. Faded examples also encourage learners (and instructors) to think about the similarities and differences between various approaches.

Worked examples are themselves an example of “concreteness fading” [38, 39], which describes the process of starting lessons with things that are specific or tangible and then explicitly and gradually transitioning to more abstract and general concepts. Concreteness fading

1. helps learners understand abstract symbols in terms of well-understood concrete objects,
2. lets them leverage personal experience to ground abstract thinking,
3. gives them a store of examples and mental images that they can fall back on when abstract symbols and reasoning fail, and
4. helps learners figure out what is specific to particular examples and what is generalizable across all problems of a certain kind.

One way to remember this strategy is the acronym PETE (Problem, Explanation, Theory, Example), which encourages instructors to

- describe an authentic problem that the lesson will solve,
- work through a solution to that problem,
- explain the general theory that underpins that solution, and
- work through a second example so that learners will understand which parts generalize.

## 8. Show how to detect, diagnose, and correct common mistakes

It is almost oxymoronic to say that learners spend a lot of their time trying to figure out what they've done wrong and fixing it: after all, if they knew and they had, they would already have moved on to the next subject. Most lessons devote little time to detecting, diagnosing, and correcting common mistakes, but doing this will accelerate learning—not least by reducing the time that learners spend feeling lost and frustrated.

In Carroll and colleagues’ “minimal manual” approach to training materials, every topic is accompanied by descriptions of symptoms learners might see, their causes, and how to correct them [40]. When studying second language acquisition, [41] identified six ways in which instructors can correct learners' mistakes:

- clearly indicating that the learner is incorrect and provide the correct form,
- repeating the learner's response with the mistake or mistakes corrected,
- indicating that the learner's answer is incorrect (e.g., by saying, "Are you sure?") but leaving the correction open-ended,
- posing leading questions (e.g., "Do we need the absolute error or the relative error here?"),
- providing the first part of the correct answer as a prompt and require the learner to fill in the rest, and
- repeating the learner's error, drawing attention to it but leaving the correction up to them.

All of these can be used preemptively during the design of lessons. An introduction to chemical reactions, e.g., could present an incomplete calculation of enthalpy and ask the learner to fill it in (elicitation) or present the complete calculation with errors, then draw attention to those errors and correct them one by one (recasting). All of these strategies provide retrieval practice by requiring learners to use what they have just learned and encourage metacognition by requiring them to reflect on the limits and applicability of that knowledge.

## 9. Motivate and avoid demotivating

One of the strongest predictors of whether people learn something is their “intrinsic motivation,” i.e., their innate desire to master the material. The term is used in contract with “extrinsic motivation,” which refers to behavior driven by rewards such as money, fame, and grades. As [42] describes, the biggest motivators for adult learners are their sense of agency (i.e., the degree to which they feel that they're in control of their lives), the utility or usefulness of what they're learning, and whether their peers are learning the same things. Letting people go through lessons at the time of their own choosing, using authentic tasks, and working in small groups speak to each of these factors.

Conversely, it is very easy for educators to demotivate their learners by being unpredictable, unfair, or indifferent. If there is no reliable relationship between effort and result, learners stop trying (a particular case of a broader phenomenon called “learned helplessness”). If the learning environment is slanted to advantage some people at the expense of others, everyone will do less well on average [43], and if the lessons make it clear that the teacher doesn't care if people learn things or not, learners will mirror that indifference.

One way to tell if learners are motivated or not is to look at the incidence of cheating. In classrooms, it is usually not a symptom of moral failing but a rational response to poorly designed incentives. As reported in [44], some things that educators do that unintentionally encourage cheating include

- setting the cost of failure very high,
- relying on single assessment mechanisms like multiple-choice tests, and
- using arbitrary grading criteria.

Eliminating these from lessons doesn't guarantee that learners won't cheat but does reduce the incidence. (And, despite what many educators believe, cheating is no more likely online than in person [45].)

## 10. Make lessons inclusive

“Inclusivity” is a policy of including people who might otherwise be excluded. In STEM education, it means making a positive effort to be more welcoming to women, under-represented racial or ethnic groups, people with various sexual orientations, the elderly, the physically challenged, the economically disadvantaged, and others.

The most important step is to stop thinking in terms of a “deficit model,” i.e., to stop thinking that the members of marginalized groups lack something and are therefore responsible for not getting ahead. Believing that puts the burden on people who already have to work harder because of the inequities they face and (not coincidentally) gives those who benefit from the current arrangements an excuse not to look at themselves too closely.

One axis of inclusive lesson design is physical: provide descriptive text for images and videos to help the visually challenged, closed captions for videos to help those with hearing challenges, and so on. Another axis is social:

- Use gender-neutral pronouns (e.g., a singular "they") or alternate between male and female pronouns.
- Use culturally varied names in examples (e.g., Aisha and Boris rather than Alice and Bob).
- Avoid examples based on oversimplified or exclusionary views of gender and orientation, such as assuming that there are only two genders, that gender is fixed throughout a person's life, or that marriage is always between people of unlike gender.

Committing fully to inclusive teaching may mean fundamentally rethinking content. [46], e.g., explored two strategies for making computing education more culturally inclusive, each of which has its own traps for the unwary. The first strategy, community representation, highlights students' social identities, histories, and community networks using afterschool mentors or role models from students' neighborhoods or activities that use community narratives and histories as a foundation for a computing project. The major risk is shallowness, e.g., using computers to build slideshows rather than do any real computing.

The second strategy, computational integration, incorporates ideas from the learner's community, e.g., by reverse engineering indigenous graphic designs in a visual programming environment. The major risk here is cultural appropriation, e.g., using practices without acknowledging origins. No matter which strategy is chosen, the first steps should always be to ask your learners and members of their community what they think you ought to do and to give them control over content and direction.

## Conclusion

Following the 10 tips laid out above doesn't guarantee that your lessons will be great, but it will help ensure that they aren't bad. When it comes time to put them into practice, we recommend following something like the reverse design process developed independently by [47, 48, 49]:

1. Figure out who your learners are and what their goals are.
2. Create the summative assessment for the lesson to give yourself a target.
3. Itemize the knowledge and skills that assessment relies on and create formative assessments to check on each while learning is taking place.
4. Order those formative assessments in a way that respects their dependencies, i.e., so that they build on each other.
5. Estimate the time required to cover each topic and perform its related formative assessment, then cut material that there isn't time for.
6. Write lessons to connect each formative assessment to the next (which is usually much easier than writing an entire lesson at once).
7. Double-check your language and examples to ensure that they address your learners' goals and won't demotivate them.
8. Derive learning objectives and key points from the lesson to share with your learners and coinstructors. The former makes the lesson findable, while the latter gives you and your coinstructors a quick way to check what the lesson actually covers.
9. Put everything online under an open license for other people to download, modify, and contribute to.

We also recommend that lessons be designed for sharing with other instructors. Instructors often scour the web for ideas, and it's common for people to inherit courses from previous instructors. What is far less common is collaborative lesson construction, i.e., people taking material, improving it, and then offering their changes back to the community. This model has served the open source software community well, and as [9] describes, it works equally well for lessons—provided that materials are designed to make fine-grained collaboration easy. Unfortunately, widely-used systems like Git are designed to handle text files and struggle with structured document formats like Microsoft Word or PowerPoint. In addition, their learning curve is very steep and deters many potential users who have deadlines to meet or would rather think about engaging exercises than try to make sense of obscure error messages.

One key enabler of collaborative lesson construction is licensing. We strongly recommend using one of the Creative Commons family of licenses since they have been carefully vetted and are widely understood.
